# Characterization of Suspected Crimes against Companion Animals in Portugal

**DOI:** 10.3390/ani11092744

**Published:** 2021-09-20

**Authors:** Diana Araújo, Carla Lima, João R. Mesquita, Irina Amorim, Cristina Ochôa

**Affiliations:** 1 Instituto de Ciências Biomédicas de Abel Salazar (ICBAS), Universidade do Porto (UP), Rua de Jorge Viterbo Ferreira, 228, 4050-313 Porto, Portugal; dianac.araujo@hotmail.com (D.A.); jrmesquita@icbas.up.pt (J.R.M.); 2 Instituto Nacional de Investigação Agrária e Veterinária, Rua dos Lagidos, Lugar da Madalena, 4485-655 Vairão, Portugal; carla.lima@iniav.pt (C.L.); cristina.ochoa@iniav.pt (C.O.); 3 Epidemiology Research Unit (EPIUnit), Instituto de Saúde Pública da Universidade do Porto, Rua das Taipas 135, 4050-600 Porto, Portugal; 4 i3S—Instituto de Investigação e Inovação da Universidade do Porto, Rua Alfredo Allen 208, 4200-135 Porto, Portugal

**Keywords:** animal abuse, forensic veterinary necropsy, dogs, cats

## Abstract

**Simple Summary:**

Crimes against companion animals are universal and represent a major problem in human/animal interaction. This study characterizes forensic cases received at the Laboratory of Pathology of the National Institute of Agrarian and Veterinary Investigation (Vairão, Portugal) since the enforcement of the Portuguese law that criminalizes the mistreatment and abandonment of companion animals. Based on the consult of 160 data files of forensic necropsies analyzed for this study, the suspicion of prior crime against companion animal was confirmed in 38 cases (24%). Most of the assaulted animals were medium-size (57%) male (58%) dogs (87%) of crossbreed (55%), whose death was related to blunt force trauma (31%), firearms (27%), poisoning (27%), and asphyxiation (15%). However, in cats, death was related to blunt force trauma only (100%). In Portugal, violence against animals is a reality and the complaint of these crimes is gradually increasing due to the population’s raising awareness about animal rights.

**Abstract:**

Animal crimes are a widespread phenomenon with serious implications for animal welfare, individual well-being and for society in general. These crimes are universal and represent a major problem in human/animal interaction. In Portugal, current law 69/2014 criminalizes the mistreatment and abandonment of companion animals. This study characterizes forensic cases received at the Laboratory of Pathology of the National Institute of Agrarian and Veterinary Investigation (Vairão) since the enforcement of the aforementioned legislation. A retrospective study was carried out based on the consult of 160 data files of forensic necropsies from 127 dogs and 33 cats. Necropsies confirmed prior crime suspicion in 38 cases (24%), from which 33 were dogs and five were cats. Among confirmed cases, most of assaulted animals were medium-size (57%), crossbreed (55%) male (58%) dogs (87%), which were the victims of blunt force trauma (31%), firearms (27%), poisoning (27%) and asphyxiation (15%). In cats, most of the assaulted animals were juvenile (60%) females (60%) of unknown breed (40%), which suffered blunt force trauma (100%) as the only cause of death. The present study shows that violence against animals is a reality, and complaints about these crimes are gradually increasing due to the population’s raising awareness about animal rights. Greater communication and coordination between clinicians, veterinary pathologists, and law enforcement officers are essential to validate and legally support these cases and subject them to trial.

## 1. Introduction

Animals are endowed with conscience and remain the most vulnerable group of all sentient beings, as they totally depend on humans to survive. Animals need care in the same way as children do, but unlike them, they do not have a voice, and will never gain independence, remaining vulnerable throughout their lives and hence subject to abuse and cruelty [1].

Animal crimes are a widespread phenomenon with serious implications for animal welfare, individual well-being, and for society in general [2]. Crimes against companion animals are universal and represent a major problem in human/animal interaction, since the main threat to animals arises from human behavior [3].

In recent decades, scientific studies have proven that animal abuse and interpersonal violence can occur simultaneously. It is now recognized that crimes against animals and domestic violence can co-exist and that animal abuse can be indicative of intrafamiliar problems [4]. The concerns and fears that influence the society’s perception on animal suffering work as a cultural filter on people’s views about cruelty [5].

Animal cruelty occurs within a social context, and it is the responsibility of the community members working in the field of animal health and safety to find resources to put an end to animal victimization, namely by promoting greater understanding of the human/animal relationship. Studies in this area also provide new perspectives on the subject of violence [6].

Investigations and prosecutions of crimes against companion animals are common occurrences and attract widespread attention. Countless animal crimes are committed every day and anywhere but rarely by disturbed individuals. The highlight of these events by the media has increased awareness in the general population about animal abuse [7].

Despite recent advances on this topic, the number and most frequent causes of animal abuse in Portugal are still unknown. The aim of the present study is to characterize canine and feline necropsy cases received at the Laboratory of Pathology of the National Institute of Agrarian and Veterinary Investigation (INIAV, Vairão, Portugal) since the implementation of the legislation criminalizing companion animal abuse in the country, namely between 29 August 2014 and 31 March 2020, and for which a suspicion of crime against companion animals already existed.

## 2. Materials and Methods

### 2.1. Sample Population and Cases Selection

A retrospective study was carried out based on the consult of the data files from the canine and feline forensic necropsies performed at the Laboratory of Pathology of the National Institute of Agrarian and Veterinary Investigation (INIAV, Vairão). The INIAV laboratories are national reference laboratories for the diagnosis of animal diseases and the Vairão subunit, which is located in the north of Portugal, mainly covers the needs of the northern coastal region despite being able to receive cases from the whole country. The laboratory performs a total of 500 necropsies per year, and its social area of intervention is mainly urban.

The cases received between 29 August 2014 (date of enforcement of the Portuguese law that criminalizes the mistreatment and abandonment of companion animals) and 31 March 2020 were selected for this study and detailed information was extracted from them regarding (1) the victim: species, age, breed, sex, clinical history/suspicion, results of forensic necropsy, and complementary diagnostic tests such as toxicology research and X-rays; (2) the crime: country region of occurrence, public location or private property, and the type of weapon used (whenever applicable); and (3) gender of complainants.

Both animal species were classified according to their age groups (as juvenile, adult, or senior), and dogs in particular were characterized according to their size (as small, medium, or large breeds), as proposed by Fred L. Metzger [8].

### 2.2. Statistical Analysis

Descriptive statistical analysis was performed using GraphPad Prism software (version 5.04).

## 3. Results

In the period under study, a total of 2981 post-mortem examinations were performed, among which 160 (5.4%) were classified as forensic necropsies and included 127 dogs (79.4%) (56 females, 69 males and two undetermined specimens due to advanced cadeveric decomposition) and 33 cats (20.6%) (14 females and 19 males).

**Figure 1:**
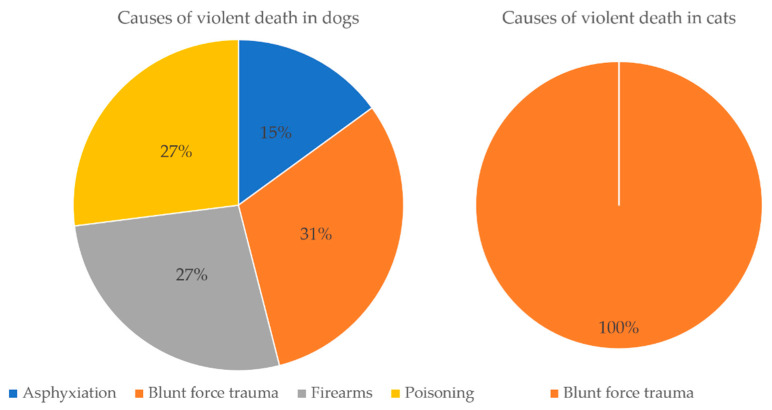
Graph representing the causes of violent death in dogs (*n* = 33) and cats (*n* = 5) in the period under study.

The forensic necropsies confirmed the prior crime suspicion in 38 cases (24.0%) (Table 1 and Table 2) (Figure 1).

In the remaining 122 cases (76.0%), death did not occur violently: 63 (39.0%) deaths resulted from natural causes or pathological conditions and in 59 cases the necropsies led to inconclusive results (37.0%). In dogs, most cases lacking a final verdict after a forensic necropsy was performed were related to suspected poisoning, negligence, or abandonment (which are complex issues themselves and are difficult to prove without resorting to other types of evidence or eyewitnesses).

Similar results were obtained in cats, for which approximately one third (36.0%) of suspected poisoning cases could not be confirmed.

### 3.1. Victim

Among the cases in which post-mortem analysis was compatible with violent death, 33 were related to dogs (87.0%) and five to cats (13.0%) (Table 1 and Table 2).

With regard to dogs, 18 animals were of crossbreed (55.0%), 15 presented a defined breed (45.0%), and only one specimen was considered as a potentially dangerous breed (XRottweiler). From these 33 dogs, 19 were male (58.0%), and 14 were female (42.0%). For the 23 dogs with known age group, nine were adults (39.0%), seven were juveniles (30.5%), and seven were seniors (30.5%); the youngest was a one-month old female drowning victim of undetermined breed; the eldest was a 10 year old crossbred male that was strangled. From the 33 dogs, it was possible to determine the size for 30 of them (91.0%); 17 were medium-sized (57.0%), nine were large-sized (30.0%), and four were small-sized (13.0%). Size could not be determined for three puppies due to their young age (1–2 months old). The average weight of dogs was 16 kg, which falls into the medium size category. For the 33 dogs whose cause of death was determined as violent, 10 were injured through blunt force trauma (31%); nine with firearms (27%); nine others through poisoning (27.0%); and five through asphyxiation (15.0%).

For cats, three out of five were female (60.0%), and two were male (40.0%) with different breeds: one was a Common European (20%), one was a Siamese (20.0%), another was an XSiamese (20.0%), and the breed could not be determined of two of them (40.0%). Three cats were juvenile (60%), one was an adult (20%), and the age group of the last one could not be determined (20%). The youngest was a three-day old male feline and the eldest an 8 year old female XSiamese. The cause of death for the five cats was related to trauma.

**Figure 2:**
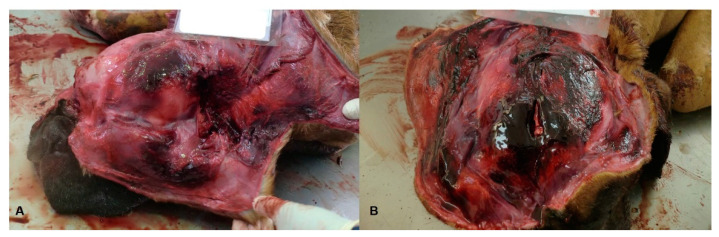
(**A**,**B**): Traumatic brain injury of blunt nature in two senior, large sized, male Boxer dogs belonging to the same owner. Traumatic brain injuries, compatible with action of a blunt object which caused compression fractures of the head, with extensive hemorrhagic lesions and neuroparenchyma tear at the level of the brainstem.

Amongst the animals victimized through blunt force trauma, three dogs (30.0%) and two cats (40.0%) suffered traumatic brain injuries (Figure 2A,B).

**Figure 3:**
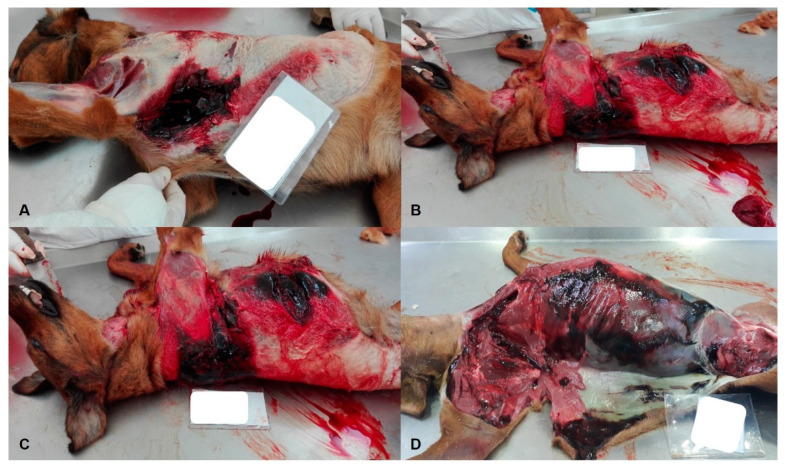
(**A**–**D**): Other injuries of blunt force trauma in dogs. Adult, medium-sized, crossbred female dog, presenting fractures of the right femur, subcutaneous sero-bloody infiltrations, with muscle haemorrhages, rib fractures, petechiae and subcutaneous suffusions in the neck and chest, and subcutaneous haemorrhagic infiltrations in the right shoulder.

Additionally, four dogs (40.0%) and one cat (20.0%) suffered motor vehicle or traffic road accidents presenting signs of polytrauma consisting of bone fractures and osteoarticular injuries, organs fractures (spleen, liver, and kidneys), internal hemorrhages, and subcutaneous and muscular bruises. As for the remaining cases of blunt force trauma, one dog (10.0%) also presented multiple fractures and hemorrhages (Figure 3A–D).

Perforating ante-mortem lesions were also observed on the right forearm and trunk of a canine apparently sick and unable to move, which was found in a ditch. Due to its extreme weakness, the animal was dragged into this location with the help of a rug, which corroborates the suspicion of abuse and abandonment. Subsequently, the animal was euthanized by the veterinarian clinician in a high suffering state.

In one dog (10.0%), death occurred due to closed chest trauma with pulmonary fractures and haemothorax, compatible with history of kicks allegedly perpetrated by an individual who later attacked another dog that survived with severe injuries and lost locomotor capacity.

As for cats, two (40.0%) suffered thoraco-abdominal trauma with subcutaneous haemorrhagic infiltrations and pulmonary and hepatic tears, compatible with previous testimonies of fall or defenestration.

**Figure 4:**
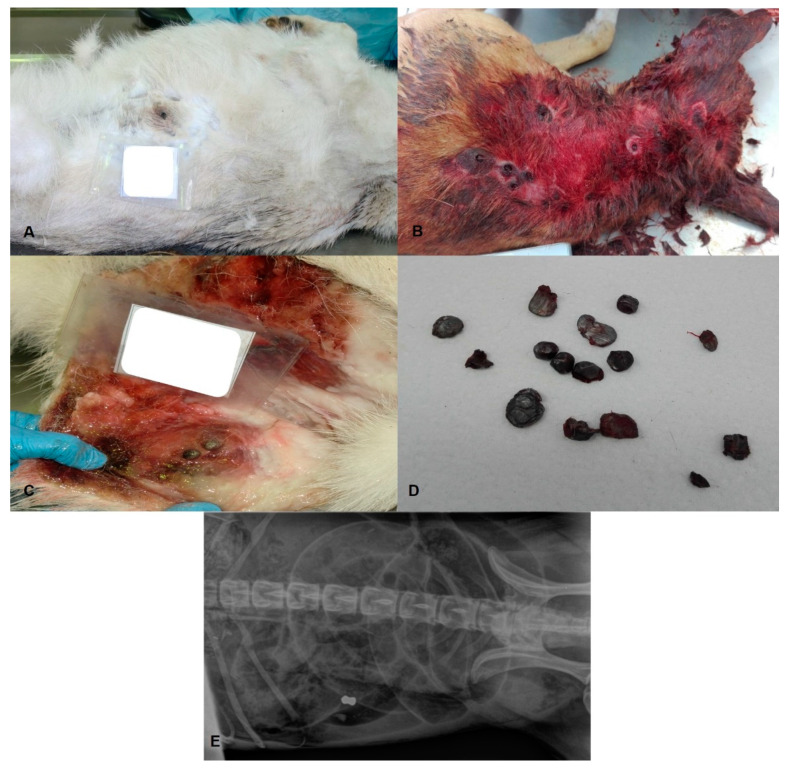
Dog. (**A**): Projectile injury caused by firearm with an exit hole in the abdomen in a senior, large-sized, female Siberian Husky; (**B**): Projectile injuries in an adult, medium-sized, male Poodle shot down during hunting; (**C**,**D**): Firearm projectiles collected at necropsy; (**E**): Radiography showing a projectile that reached the intestine in a medium-sized, male Poodle.

In this investigation, injuries caused by firearms reached the thoraco-abdominal region in four cases (45.0%) and the thoracic region only in three cases (33.0%) (Figure 4A,B), causing, in both regions, internal bleeding, bone fractures, and injuries to various organs, namely to the heart, lungs, liver, spleen, and intestine. In one out of nine cases (11.0%), only the abdominal region was affected, with internal bleeding occurring through perforation of the abdominal aorta. The head region was also affected in one case (11.0%) with fracture of the temporal and parietal cranial bones, fracture near the atlanto-occipital joint, and brainstem and cervical cord laceration of the neuroparenchyma by lead spheres. The projectiles from these cases were collected for ballistic examination (Figure 4C,D) and five of these cases were also subjected to imaging examination, namely X-ray, before the necropsy (56.0%) (Figure 4E).

**Figure 5:**
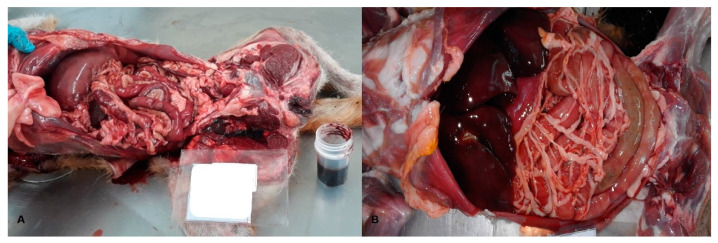
Generalized congestive-hemorrhagic conditions diagnosed post-mortem in dogs. (**A**): Medium-sized, female Poodle poisoned with carbamates; (**B**): Juvenile, female crossbreed poisoned with carbamates.

In the present study, canine poisonings occurred due to carbamates (45%), followed by rodenticides (33.0%), organophosphates (11.0%), and cyanide (11.0%). In five out of nine of the poisoning cases, the macroscopic findings corresponded to generalized congestive-hemorrhagic conditions (56.0%) (Figure 5A,B).

In two of these cases, the cause of death was not determined at necropsy (22.0%), and in the remaining two cases, the necropsy result was inconclusive due to advanced cadaveric decomposition (22.0%). However, in all of them, the toxicological tests confirmed poisoning.

**Figure 6:**
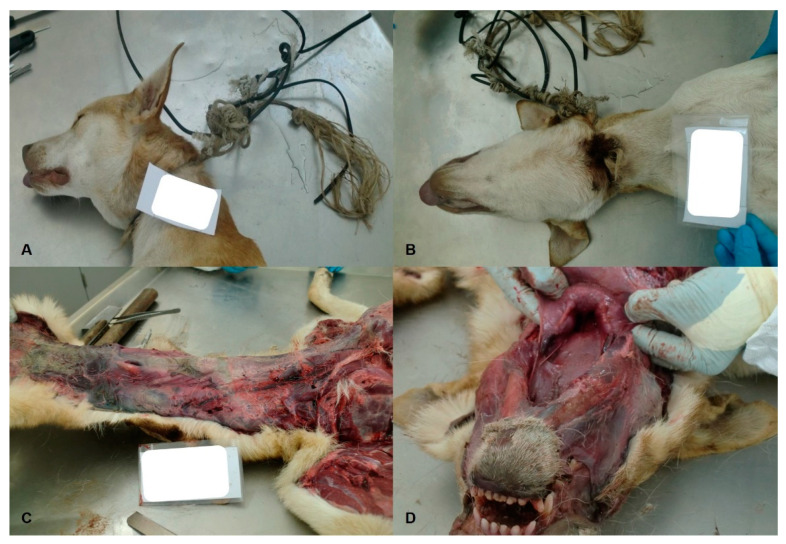
Loop strangulation (**A**) and groove with lacerating wound (**B**) in a dog. Hemorrhages in the neck (**C**) without loss of integrity of the hyoid bones (**D**) in an adult, crossbreed, male and medium-sized dog, hanged by rope in a tree, with incomplete suspension of the body, hanging through the lap area, with the hips and feet resting on the floor.

Regarding the 15.0% of asphyxiation cases, four were due to strangulation (80.0%) (Figure 6A,B). In one of these animals, necropsy findings were compatible with hanging through incomplete body suspension (Figure 6C,D). This animal exhibited congestion of the head area, exophthalmos, mark of the incomplete groove in the mandibular coat, hemorrhages in the soft tissues of the neck, larynx, and peritraqueal, blackened blood coloring, tracheobronchial foam, and pericardium hemorrhages.

Another asphyxiated animal was found in a sewer pipe in the streets, presenting signs of drowning (20.0%). Macroscopically, the lungs were enlarged, inflated in aspect, crackling on palpation with multifocal to coalescent and miliary subpleural bright red lesions, which were more extensive at the periphery of the lung edges, with blood and foam exudation at cross section.

### 3.2. Crime Geographical Location and Context

**Figure 7:**
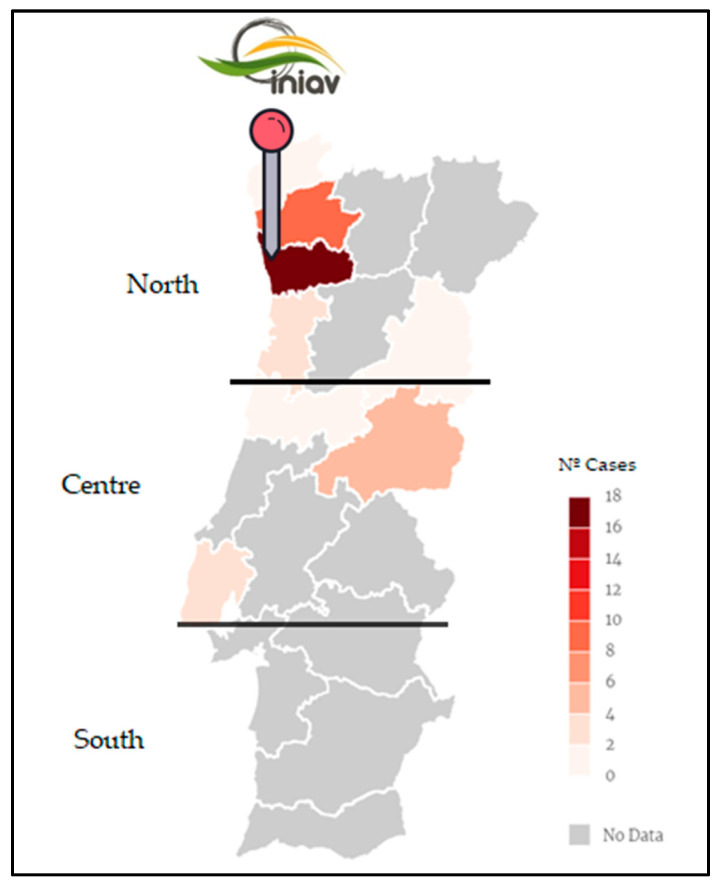
Geographical representation of the location of INIAV reference laboratory and the origin of the forensic cases included in this study.

From the 38 confirmed cases, 30 occurred in the northern region of Portugal (79%) and eight in the central region (21%) (Figure 7).

Among the total crimes committed with firearms, three occurred in a public location (33.5%), two inside a private property (22.0%), three during hunting activities (33.5%), and this information is unknown for the remaining cases (11%).

### 3.3. Complainant and Complaints

In 22 out of 26 (85.0%) reports of suspected crimes against companion animals, it was possible to retrieve information about the gender of the complainant: 11 were men (50.0%) and 11 were women (50%).

In 2014, there were no reports of these crimes, and in 2015, there was only one criminal complaint. However, a growing number of complaints was noticed from 2016, which culminated in 20 criminal complaints in 2019. Despite the increase in reported cases, the average number of violent death cases confirmed through forensic necropsy remained stable per year.

## 4. Discussion

During the period under study, 160 suspected cases of crimes against companion animals were admitted to the Laboratory of Pathology of INIAV Vairão, but the forensic necropsies only confirmed the suspicion of violent death in less than a quarter.

Similarly to previous studies, dogs are the most affected species, followed by cats [9]. The tendency to commit crimes against a specific animal species can be explained by several factors, namely the availability and behavior of the animal species, the social attitude towards them, and their physical characteristics determining adequacy for abuse. Cats, in comparison to dogs, are more independent, have more free access, and spend more time outside and, most often, do not have a known owner. On the other hand, some dogs are more empathetic and submissive animals, more dependent on human interaction, and maintain these behaviors even when they are the victims of abuse [10].

In the present study, the small number of formalized cases against cats can be explained by the inherent behavior of the species; when cats are injured or frightened, they tend to isolate and hide themselves and avoid human interaction, making it difficult to be detected as victims of abuse. In addition, the owner may delay the search for his missing cat, thinking that it will eventually return or find a new home. Negative social attitudes towards cats can potentially influence public opinion making criminal acts against this species significantly underreported [11].

Although the number of males is higher than females, no major differences were found in the present study. According to Intarapanich et al. (2016), male animals were more frequently the victims of non-accidental injuries and motor vehicle accidents; however, this difference was not statistically significant [10]. In another report, male dogs were probably more affected by violent acts, due to the fact that they can behave more aggressively and less controllably than females, or because aggressors tend to prefer male animals [12].

The age distribution of animals suffering violent death follows a normal distribution, showing no evidence of a certain age group preference. However, several authors claim that animal victims tend to be young [10,12,13]. Young animals are more immature, restless, and difficult to control, and hence probably disturb and irritate their owners, triggering aggressive behaviors from them. Young animals are also more fragile and less able to defend themselves or escape [10,12,13]. Additionally, younger animals tend to explore new environments, being more susceptible to criminal acts by neighbors, for example [12].

Most affected dogs are medium-sized, which are normally easier to control and manipulate by humans, followed by large-sized animals. Some studies show that large-sized dogs are more affected since they are more likely to be kept outside the house and remain more exposed to the observation of the general population who may denounce a suspicion of mistreatment [14,15].

Blunt force injuries are frequent findings in veterinary forensics. Lesions in companion animals are comparable with those described in humans and crucial information can be obtained through macroscopic and microscopic examinations in order to provide evidence to court cases [16]. In the present study, blunt force trauma was found to be a major cause of violent death in dogs and cats. Violent death can arise from accidental or non-accidental causes and vary from: motor vehicle accidents; falls from heights; activity injuries; and injuries resulting from physical aggression. It is essential to examine the entire animal in order to ascertain the incident and events sequency; there may be signs of repetitive abuse, such as bruises, scars, or multiple fractures revealed at different stages of convalescence and indicative of occurrence at different time points [17].

The importance of the injury depends on its anatomical location and on the size and nature of the traumatic pathological process. In the present study, 30% of dogs and 40% of cats presented traumatic brain injuries. Lesions affecting the CNS are more likely to culminate in death [18]. Animals wounded on public roads can be run over or suffer accidents by motor vehicles [13], and dogs seem to be more involved in these accidents than cats [10].

In the present study, violent deaths provoked by projectiles victimized only dogs. Capak et al. (2016) reported that, annually, approximately 42 dogs and 28 cats suffer projectile injuries. This discrepancy can be explained by the fact that dogs are the most represented species in small animal veterinary clinics, emphasizing the clinical relevance of this type of lesions, especially in this species [19].

In the present study, the majority of the cases associated with firearm occurred on the public road or inside a property and a minor number during hunting activities. Similar observations were noted by Capak et al. (2016), where hunting accidents represented only 13% of projectile injuries, concluding that such lesions are a relevant cause of trauma in animals that are not used for hunting [19].

Poisonings are a serious cause of mortality in companion animals [20,21]. In the present study, cases of poisoning were found in dogs, which corroborates previous descriptions from Berny et al. (2010) in five European countries, namely in Belgium, France, Greece, Italy, and Spain. According to the Laboratory of Toxicology of Ghent University, dogs accounted for 20% of all cases of intoxication, followed by cats (11%). Similarly, in France, dogs account for 35% of the cases registered annually [22], and this value is consistent with the numbers recorded in Germany, Italy, Spain, and Austria [23,24,25,26,27]. In the USA, the Animal Poison Control Center (APCC) receives thousands of suspected cases annually. Between 2002 and 2010, 76% of the incidents received affected dogs and 13% cats [28], and this trend continued in following years as canids accounted for 65% and 63% of the cases, respectively, in 2016 and 2017 [29]. In Brazil, intoxications follow the same pattern, amounting to 86% in dogs and only 13% in cats [30]. This difference may be related to the fact that cats are more selective in their food since they tend to absolutely reject any food that presents a smell or taste that they do not find pleasant. Dogs are usually curious animals that eat and play with many things they find and have a voracious appetite [21,30,31].

The most common substances involved in intoxications are insecticides, rodenticides, other pesticides, such as herbicides and fungicides, plants, human and veterinary drugs, metals, household products, toxins, and ingredients that comprise human food [22,26,28,29,32]. Most poisonings occurred through carbamates (45%), rodenticides (19–33%), and organophosphates (5–11%) [22,25,30]. According to Guzmán et al. (2002), the high rates of insecticide poisoning are due to their widespread use in agricultural practices [25]. In the present study, only one death occurred due to cyanide poisoning. This can be explained by the use of this product in the jewelry industry [33,34,35], since the animal in question came from an area of the country where this activity is prevalent.

The main causes of poisoning in urban environments result from interpersonal conflicts. However, in rural areas, these cases arise in the course of human activities, such as hunting or agriculture [13,23,36,37]. The production of poisonous baits may not be difficult, since some of the agents used for this purpose are easily accessible on the market and the choice of a specific toxicant for intentional animal poisoning may arise from popular knowledge about its toxicity and commercial availability [38]. Intoxications depend on various available features in the animals’ environment: the tendency of exposure of the animal to the agent; the toxin amount and also the individual sensitivity of the animal to the toxic agent adverse effects [20]. Epidemiological studies are scarce, and the actual number of intoxication cases may be underestimated. This fact comes from the animals’ natural inclination to hide when they feel sick, making it difficult for their body to be recovered after death [23,25]. Another fundamental fact is the failure in clinical or analytical diagnosis, since tests are often not performed because there is no toxicological screening capable of detecting all known toxic agents. Thus, in addition to being expensive, tests can be unsuccessful [37]. These factors may have influenced the number of poisoning cases in the present study as the necropsy often leads to inconclusive diagnosis either due to cadaveric decomposition or to nonspecific congestive-hemorrhagic conditions and without toxicological tests being performed, the suspicion cannot be confirmed and cannot be excluded either.

In the present study, 15% of deaths occurred due to asphyxiation and only dogs were the victims of this type of act [12]. It is plausible that an animal could die as a result of accidental hanging with a rope due to incomplete suspension [39]. It is known, however, that some people practice the execrable act of drowning new-born animals in order to get rid of them. The frequency of animal drowning is unknown, and the literature addressing this topic is sparse [40,41]. A drowning diagnosis in animals is even more complex than in humans due to the different anatomophysiological respiratory system specificities of the species, and it remains a challenge both for human and veterinary forensics [42]. There are no pathognomonic drowning necropsy findings; therefore, drowning remains an exclusion diagnosis based on the circumstances of death and nonspecific findings of the necropsy [43]. As in humans, in suspected cases, the presence of diatoms can be investigated [42], although this was not found necessary in the only case present in this study.

The largest number of cases are located in the northern region of Portugal, but this does not necessarily mean that more criminal acts are committed there. This can simply indicate that the number of animals is higher, or that the population is more likely to report these cases in this geographical location. Moreover, the Vairão subunit of INIAV where this study took place is also based in this region. Therefore, most of the complaints were focused on northern coastal districts, which present marked rural and urban contrast. This geographical distinction, which only allowed us to study one location by country region, largely limits our interpretation of the real social context.

No statistical differences were found between the complainants’ genders in this study. However, studies revealed a greater propensity of female individuals to report this type of crime, varying between 40% to 80%, while male individuals vary between 12% and 22% [9,14,15]. Previous studies demonstrated that men who witnessed acts of animal cruelty showed a more insensitive attitude, while women exhibited greater sensitivity and seemed to be more emotionally linked to companion animals [44,45].

The annual evolution in the number of cases with a necropsy conclusion compatible with crime shows that before 2016 the number of these cases was inferior, at five per year. After that, this number approximately doubled. This may be related to the election of a specific Portuguese political party in 2015 with well-defined aims on animal welfare. The greater expression and verbalization of this political party in society may have given confidence to some citizens to report their suspicions of crimes against companion animals, which may also reflect the number of cases confirmed as a criminal act. This increase over the years does not necessarily mean that there is more violence against animals than in previous years. It can merely suggest that people have become more sensitive to the topic and therefore are more likely to report their suspicions.

McEwen (2012) found that there is a gradual linear trend, which is consistent and statistically significant, in the submission of cases for forensic necropsy. Several reasons can justify this propensity, such as an increasing attention from the media, changes in the country’s justice system and legislation, the relationship between crimes against companion animals and domestic violence, as well as professional and public interest [46]. Even so, from all the suspected cases submitted in the period under analysis, approximately 37% proved to be inconclusive after the forensic necropsy, and the vast majority are related to the advanced decomposition state of the corpse, which makes it impossible to carry out most of the necessary tests, and to limitations related to the laboratory resources (as certain cases may require specific tests that are not included in the conventional routine of the official laboratory).

The difficulty in producing clear evidence, due to lack of proper examination of the crime scene and scarce laboratory and economic resources, supported by the existence of unclear legislation that is difficult to interpret, mean that gathered facts are not sufficient to prove the crime or the author of the criminal act.

## 5. Conclusions

The present retrospective study has allowed the identification of suspected cases of crimes against companion animals by investigating the main causes of violent death through the characterization of animals/victims and to evaluate injuries found in forensic necropsies.

Companion animals play an important role in the life of human beings. The reports of crimes against companion animals have clearly increased in recent years; however, this may not be directly indicative of a real increase in abuse acts. We must concede that the growing awareness of the society regarding animal rights translates into the civic duty of reporting abuses in order to guarantee animal protection. Still, we believe that there are many more crimes than those reported and only a small percentage reach the courts and are judged, and an even smaller percentage are condemned.

In order to move safely towards an evolved and fair society, the training of professionals in this area is crucial. Law enforcement officers and veterinarians must have adequate training, share knowledge, and work together to obtain legally valid evidence. In Portugal, although greater sensitivity, interest, and media coverage of animal violence is noticeable, there is still a long way to go.

## Figures and Tables

**Table 1:** Characterization of the canine cases of violent death confirmed through forensic necropsy.

YEAR Total = 33	BREED	SEX	AGE	ANIMAL SIZE ^1^	CAUSE OF DEATH	COMPLEMENTARY EXAMS	CRIME SCENE DETAILS (*n*/%)	
	(*n*/%)	(*n*/%)		(*n*/%)	(*n*/%)	(*n*/%)	Country Region	Context
2014 *	Poodle (1/3.0)	F (1/3.0)	J (1/3.0)	m (1/3.0)	Poisoning (1/3.0)	Toxicology (1/3.0)	North (1/3.0)	NA
*n* = 1								
2015	Crossbreed (2/6.0)	F (1/3.0)	J (1/3.0)	m (1/3.0)	Asphyxiation (2/6.1)	-	North (1/3.0)	NA
*n* = 2		M (1/3.0)	S (1/3.0)	U (1/3.0)			South (1/3.0)	
2016	Labrador (1/3.0)	F (1/3.0)	A (1/3.0)	l (4/12.0)	Blunt force trauma (4/12.0)	-	North (4/12.0)	NA
*n* = 4	Greyhound (1/3.0)	M (3/9.0)	S (3/9.0)					
	Boxer (2/6.0)							
2017	Poodle (3/9.0)	F (5/15.0)	J (3/6.0)	s (1/3.0)	Blunt force trauma (3/9.0)	X-ray (2/6.0)	North (6/18.0)	Public access (3/9.0)
*n* = 9	XRottweiller (1/3.0)	M (4/12.0)	A (5/15.0)	m (5/15.0)	Firearm (3/9.0)	Toxicology (1/3.0)	Centre (3/9.0)	
	Crossbreed (5/15.0)		S (1/3.0)	l (1/3.0)	Poisoning (3/9.0)			
				U (2/6.0)				
2018	Poodle (1/3.0)	F (2/6.0)	J (1/3.0)	s (2/6.0)	Asphyxiation (2/6.0)	X-ray (2/6.0)	North (7/21.0)	Public access (1/3.0)
*n* = 8	Siberian husky (1/3.0)	M (6/18.0)	A (3/9.0)	m (5/15.0)	Firearm (2/6.0)	Toxicology (4/12.0)	Centre (1/3.0)	
	Crossbreed (6/15.0)		S (1/3.0)	l (1/3.0)	Poisoning (4/12.0)			
			U (3/9.0)					
2019	Poodle (3/9.0)	F (3/9.0)	J (2/6.0)	s (1/3.0)	Asphyxiation (1/3.0)	X-ray (1/3.0)	North (5/15.0)	Private property (2/6.0)
*n* = 7	XFila S. Miguel (1/3.0)	M (4/12.0)	S (1/3.0)	m (5/15.0)	Blunt force trauma (3/9.0)	Toxicology (1/3.0)	Centre (2/6.0)	
	Crossbreed (3/9.0)		U (4/12.0)	l (1/3.0)	Firearm (2/6.0)			
					Poisoning (1/3.0)			
2020 **	Crossbreed (2/6.0)	F (1/3.0)	U (2/6.0)	l (2/6.0)	Firearm (2/6.0)	-	North (2/6.0)	Public access (2/6.0)
*n* = 2		M (1/3.0)						

* Only from August to December 2014. ** Only from January to March 2020. Sex: F, female; M, male. Age: J, juvenile; A. adult; S, senior; U, undetermined. ^1^ According to the criteria proposed by Fred L. Metzger [8]: s, small; m, medium; l, large; U, undetermined; -, not performed; NA, not available.

**Table 2:** Characterization of the feline cases of violent death confirmed through forensic necropsy.

YEAR Total = 5	BREED	SEX	AGE	CAUSE OF DEATH	CRIME SCENE DETAILS (*n*/%)	
	(*n*/%)	(*n*/%)	(*n*/%)	(*n*/%)	Country Region	Context
2014 *	Common european (1/20.0)	F (1/20.0)	J (1/20.0)	Blunt force trauma (1/20.0)	North (1/20.0)	NA
*n* = 1						
2015	XSiamese (1/20.0)	F (1/20.0)	A (1/20.0)	Blunt force trauma (1/20.0)	North (1/20.0)	NA
*n* = 1						
2016	Siamese (1/20.0)	F (1/20.0)	U (1/20.0)	Blunt force trauma (1/20.0)	Centre (1/20.0)	NA
*n* = 1						
2019	ID (2/40.0)	M (2/40.0)	J (2/40.0)	Blunt force trauma (2/40.0)	North (2/40.0)	NA
*n* = 2						

* Only from August to December 2014. ID, impossible to determine. Sex: F, female; M, male. Age: J, juvenile; A, adult; S, senior, U, undetermined. NA, not available.
